# 1672. Applying Spatial Analyses to Identify Pediatric Risk Factors for Recurrent *Staphylococcal aureus* Skin and Soft Tissue Infections

**DOI:** 10.1093/ofid/ofad500.1505

**Published:** 2023-11-27

**Authors:** Daria Anderson, Xiting Lin, Noor Mohamed, Lilly Immergluck, Kevin Thornton, Langdon Sanders

**Affiliations:** Morehouse School of Medicine, Atlanta, Georgia; Morehouse School of Medicine, Atlanta, Georgia; Morehouse School of Medicine, Atlanta, Georgia; Morehouse School of Medicine, Atlanta, Georgia; Morehouse School of Medicine, Atlanta, Georgia; Georgia Institute of Technology, Atlanta, Georgia

## Abstract

**Background:**

Community onset *Staphylococcus aureus* (*S. aureus*) disproportionately cause skin and soft tissue infections (SSTI) among African Americans (AA). Recurrence of *S. aureus* SSTI can be seen in >50% of pediatric cases. Risk factors associated with recurrent infections in children are not well reported. *We hypothesize that recurrence of S. aureus SSTI in AA children living in Atlanta, Georgia may be related to location-based risks.*

**Methods:**

Patients with *S. aureus* infections from 2011-2019 were included in this retrospective study, using electronic health records (EHR). We included patients who had community-onset *S. aureus* SSTI, were < 19 years old, and were Georgia resident. Sociodemographic variables collected included race, ethnicity, age, sex, and health insurance. Area-level variables from US Census Bureau included proxies for poverty, education, unemployment, and household crowding. Infections were stratified into MRSA and MSSA by oxacillin susceptibility. Recurrent SSTI was defined as an initial SSTI with one or more subsequent SSTI documented more than 14 days from initial infection within a calendar year. Home address was geocoded and assigned a census tract. Statistical analyses included chi-squared tests and multiple logistic regression to detect differences between patients with recurrent SSTI from non-recurrent SSTI using SPSS. Hotspot analyses were performed at the census tract level to identify hot and cold spots for recurrent SSTI stratified by methicillin susceptibility using ArcGIS Pro.

**Results:**

5,784 unique children presented with *S. aureus* SSTIs; 64 patients had recurrent SSTI (42 MRSA and 22 MSSA). Females had high proportion (64.1% ) of recurrent SSTIs compared to 48.0% of females with single SSTI (p = 0.011). Females also were significantly more likely to have recurrent MSSA (81.8%) compared to 54.8% of females with recurrent MRSA (p = 0.032). Spatial analysis demonstrated hotspots of recurrent SSTIs in the northern counties of metropolitan Atlanta, whereas single episode SSTIs did not demonstrate this type of spatial distribution.

SSTI Recurrence by County Level

**Figure:**
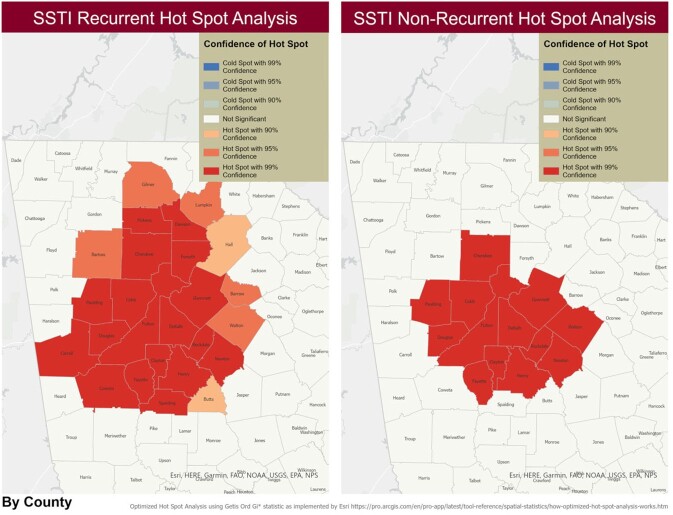

**Conclusion:**

In Georgia, children who are female are at increased risk for recurrent SSTI, and hotspots of patients with recurrent SSTIs are distinct from those with single episodes of SSTIs.

**Disclosures:**

**Lilly Immergluck, MD,MS**, Melinta: Grant/Research Support|Melinta: Grant/Research Support

